# A Rare and Isolated Presentation of Primary Cutaneous Anaplastic Large Cell Lymphoma on the Breast

**DOI:** 10.7759/cureus.49387

**Published:** 2023-11-25

**Authors:** Khulood Almarzooqi, Noura Almarzooqi, Zaidoon Abdelhadi

**Affiliations:** 1 Dermatology, Sheikh Khalifa Medical City, Abu Dhabi, ARE; 2 Internal Medicine, Sheikh Khalifa Medical City, Abu Dhabi, ARE

**Keywords:** cutaneous lymphoma, lymphomatoid papulosis, ctcl: cutaneous t-cell lymphoma, primary cutaneous t-cell lymphoma, t-cell lymphomas, t cell, breast, t-cell cutaneous, primary cutaneous anaplastic large cell lymphoma, anaplastic lymphoma

## Abstract

Primary cutaneous anaplastic large-cell lymphoma (PC-ALCL) is a subtype of non-Hodgkin lymphoma belonging to the CD30+ spectrum of lymphoproliferative disorders. It constitutes the second most prevalent category within cutaneous T-cell lymphomas (CTCL), encompassing approximately 25% of cases. This disorder is characterized by its exclusive cutaneous involvement and favorable overall prognosis. Patients typically present with reddish-brown nodules, which may evolve into ulcers. Although some cases experience regression, complete resolution is uncommon. While most lesions manifest on the extremities, followed by the head and neck, the breast region may rarely be affected by PC-ALCL. Distinctions between anaplastic lymphoma kinase (ALK)-positive and ALK-negative subtypes have been documented in breast presentations, often associated with breast implants. In this context, we present an isolated PC-ALCL instance in a 26-year-old woman with no history of breast implants.

## Introduction

Primary cutaneous anaplastic large-cell lymphoma (PC-ALCL) is a subtype of non-Hodgkin lymphoma belonging to the CD30+ spectrum of lymphoproliferative disorders. It constitutes the second most prevalent category within cutaneous T-cell lymphomas (CTCL), encompassing approximately 25% of cases [1]. This disorder is characterized by its exclusive cutaneous involvement and favorable overall prognosis. Patients typically present with reddish-brown nodules, which may evolve into ulcers. Although some cases experience regression, complete resolution is uncommon. While most lesions manifest on the extremities, followed by the head and neck, the breast region may rarely be affected by PC-ALCL. Distinctions between anaplastic lymphoma kinase (ALK)-positive and ALK-negative subtypes have been documented in breast presentations, often associated with breast implants [2]. In this context, we present an isolated PC-ALCL instance in a 26-year-old woman with no history of breast implants.

## Case presentation

**Figure 1:**
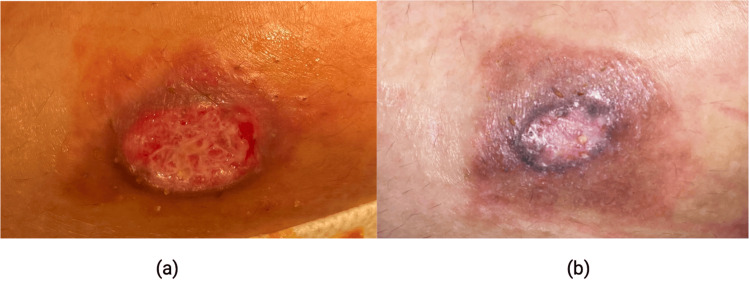
Solitary red ulcerated nodule located on the right breast (a); prior to surgical excision (b)

A 26-year-old woman presented to the dermatology clinic with evolving skin changes on her right breast. Initially, a round erythematous papule emerged just above the inframammary line. Over two months, the lesion transformed into an ulcerated nodule with adjacent erythema and bordering hyperpigmentation. Despite applying topical steroids and antibiotics, the lesion continued to enlarge over an additional three-month period, becoming friable and prone to minimal trauma-induced bleeding. No pain or pruritus was reported. Her medical history encompassed hypothyroidism and polycystic ovarian syndrome, with no prior breast-related issues or surgeries. The patient denied systemic symptoms. Physical examination revealed a solitary ulcerated nodule with violaceous margins and hyperpigmentation, measuring 1.5 x 1.5 cm as seen in Figure 1. Initially suspected as pyoderma gangrenosum due to its progression, a biopsy was performed, revealing features consistent with primary cutaneous ALCL (PC-ALCL).

**Figure 2:**
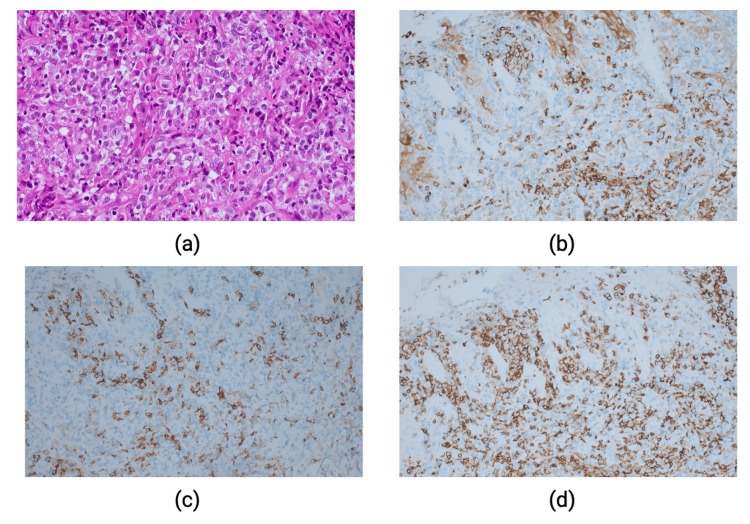
Histologic sections showing extensive infiltration by large, pleomorphic anaplastic lymphoid cells (a); immunohistochemical studies reveal that neoplastic cells are positive for EMA (b), CD30 (c), and CD43 (d).

As displayed in Figure 2, histological examination showed an acanthotic epidermis alongside a cohesive, sheet-like expansion of atypical mononuclear cells, predominantly in the mid to deep dermis, accompanied by irregular squamous epithelial strands within deeper sections. The lesion’s cells were characterized by round to epithelioid morphology, featuring enlarged nuclei, prominent nucleoli, and ample amphophilic to clear cytoplasm. An inflammatory component composed of neutrophils and lymphocytes was noted. High mitotic activity was apparent, evidenced by an 80%-90% Ki-67 proliferative index.

Abnormal cells displayed positive immunoreactivity for CD43, EMA, and CD30 while staining negative for CAM 5.2, CK7, CK20, S100, CD34, SMA, desmin, CD117, CD31, myeloperoxidase, ALK, CD3, CD20, CD68, CD23, CD21, CD1a, and lysozyme. In view of the exuberant squamous proliferation, CK5/6 and p63 stains were performed to establish if the atypical cells were also of a squamous nature. They were negative for these markers confirming that the squamous proliferation was reactive. The nature of the atypical cell proliferation was then further considered and other differential diagnoses entertained were some forms of CD30-positive lymphoproliferative disorder, cutaneous involvement by B cell lymphoma, and myeloid sarcoma. Myeloperoxidase, CD43, and CD117 stains ruled out myeloid sarcoma. CD20 stain ruled out involvement by a B cell lymphoma. The strong positivity for CD30 with negativity for the aforementioned markers was in favor of a diagnosis of CD30-positive lymphoproliferative disorder.

Based on these findings, the patient was referred to oncology for follow-up and ultimately underwent surgical excision at five months after ruling out systemic involvement through a positron emission tomography (PET) scan and laboratory blood tests. Those tests included complete blood count, peripheral blood smear, liver enzymes, complete metabolic panel, lactate dehydrogenase, and uric acid, which were all within normal limits. Flow cytometry showed 43% of T-cells with mixed T-cell subsets, thus a T-cell receptor gene rearrangement study was ordered, which was fortunately negative. Because systemic involvement was ruled out, the patient’s condition was classified as T1aN0M0 (solitary skin involvement ≤ 5 cm in diameter) according to the TNM (tumor node metastasis) classification system for cutaneous lymphomas. On future follow-ups, the patient remained asymptomatic; no constitutional symptoms nor new skin lesions were reported.

## Discussion

PC-ALCL, a subset of CD30-positive cutaneous lymphoproliferative disorders, accounts for approximately 9% of all CTCLs [3]. ALK-positive and ALK-negative presentations are rare but can affect the breast in either gender, often associated with breast implants. However, scarce literature reports cases like ours, devoid of implant history, emphasizing the potential for idiopathic occurrences [4].

Owing to its rarity and difficulty in distinguishing cases of PC-ALCL from other entities within the group of CD30-positive cutaneous lymphoproliferative disorders, the exact incidence is unknown [5]. In an analysis of the National Cancer Institute Surveillance, Epidemiology, and End Results (SEER) database, a total of 501 patients documented from 2005 to 2016 had primary localized CD30-positive cutaneous lymphoproliferative disorder. Among those cases, the median age at diagnosis was 59 years (range 2 to 97 years). Furthermore, the male-to-female ratio was 1.42 [5].

Anaplastic large-cell lymphoma (ALCL) of T-cell origin can clinically mimic many benign inflammatory skin disorders, posing a diagnostic challenge. Distinguishing it from lymphomatoid papulosis (LyP) subtypes and borderline cases relies on clinicopathologic correlation and longitudinal observation. PC-ALCL, like in our case, usually presents with solitary or multiple grouped firm nodules. On the other hand, LyP lesions present with multiple papulonodules that are much smaller in size compared to PC-ALCL and disappear spontaneously within 3 to 12 weeks [6].

Histologically, PC-ALCL is diagnosed based on the presence of diffuse infiltrates of cohesive sheets of large CD30-positive cells in >75% of the neoplastic cells. The neoplastic cells often express a CD4-positive T-cell phenotype with variable expression or loss of CD2, CD3, and CD5 [7]. Moreover, ALCL classification involves anaplastic morphology, ALK gene translocation, and CD30/ALK protein expression [8]. Although ALK-negative ALCL does not guarantee primary cutaneous disease, accurate diagnosis necessitates thorough staging investigations [9]. Prognostic implications of ALK expression are still not well-defined in cases of PC-ALCL, as many reported cases have demonstrated eventual progression to systemic involvement [10], thus cautious evaluation and treatment are essential. While not studied in our case, rearrangements of the DUSP22/IRF4 locus on chromosome 6p25.3 are found almost exclusively in PC-ALCL accounting for 20-58% of the cases, and are indicative of a favorable prognosis [7].

Characteristically, the disease presents as localized skin nodules, often ulcerated and reddish-brown. Patients presenting with an isolated lesion or a few localized nodules are initially treated through surgical excision or radiation, with subsequent therapy for recurrences [9]. The role of systemic chemotherapy has been debated, it is only indicated for extracutaneous tumor spread beyond regional lymph nodes and should be avoided in most patients with primary skin involvement [9]. Brentuximab vedotin, an anti-CD30 antibody, is offered as first-line therapy along with surgical excision in patients with concomitant involvement of skin and regional lymph nodes [11]; it is also recommended in relapsed or refractory cases, as it is proven to have positive responses in a large study [12].

Fortunately, PC-ALCL carries a favorable prognosis with a 5-year survival rate of over 90% [3]. Like other medical conditions, the outcome of PC-ALCL depends on multiple factors, including demographics, location of lesion, staging, and treatment method used. It was found that older age and extensive limb involvement adversely affect prognosis [13]. On the other hand, patients with T1 disease, a solitary lesion, or lesions showing spontaneous regression may have a more favorable course [13]. While partial regression occurs in 50% of cases, the risk of recurrence or relapse in primary ALCL is relatively high, accounting for 41% of the cases, thus follow-up is crucial for all patients [14].

## Conclusions

Our case underscores the occurrence of PC-ALCL in patients lacking identifiable risk factors. Unlike common cases linked to breast implants, our patient had no such history. We advocate for increased literature coverage to enhance dermatologists' global awareness of this pathology.
